# Reviewing the science on 50 years of conservation: Knowledge production biases and lessons for practice

**DOI:** 10.1007/s13280-024-02049-w

**Published:** 2024-07-18

**Authors:** Neil M. Dawson, Brendan Coolsaet, Aditi Bhardwaj, David Brown, Bosco Lliso, Jacqueline Loos, Laura Mannocci, Adrian Martin, Malena Oliva, Unai Pascual, Pasang Sherpa, Thomas Worsdell

**Affiliations:** 1 Global Environmental Justice Research Group, School of Global Development, University of East Anglia, Norwich Research Park, Norwich, NR4 7TJ UK; 2 Centre for the Synthesis and Analysis of Biodiversity (CESAB), French Foundation for Research on Biodiversity (FRB), 34000 Montpellier, France; 3 Fund for Scientific Research (FNRS), 1000 Brussels, Belgium; 4 Institute for the Analysis of Change in Contemporary and Historical Societies, UCLouvain, 1348 Louvain-la-Neuve, Belgium; 5 Tata Institute of Social Sciences, Mumbai, India; 6 Centre for Landscape Regeneration, University of Cambridge Conservation Research Institute, Cambridge, CB2 3QZ UK; 7 World Benchmarking Alliance, 1012 TM Amsterdam, The Netherlands; 8 Basque Centre for Climate Change, 48940 Leioa, Spain; 9 Department of Botany and Biodiversity Research, University of Vienna, 1030 Vienna, Austria; 10 Institute of Ecology and Social-Ecological Systems Institute, Leuphana University, 21335 Lüneburg, Germany; 11 MARBEC (Univ Montpellier, CNRS, Ifremer, IRD), 34070 Montpellier, France; 12 Laboratorio Nacional de Ciencias de la Sostenibilidad, LANCIS, Instituto de Ecología, Universidad Nacional Autónoma de México, Mexico City, Mexico; 13 Ikerbasque, Basque Foundation for Science, Plaza Euskadi 5, 48009 Bilbao, Spain; 14 Central Department of Sociology, Tribhuvan University, Kirtipur, Kathmandu, 44618 Nepal; 15 Amazon Frontlines, Lago Agrio, Ecuador

**Keywords:** Conservation effectiveness, Conservation science, Equitable governance, Indigenous Peoples and local communities, Participation, Rights-based conservation

## Abstract

Drawing on 662 studies from 102 countries, we present a systematic review of published empirical studies about site-level biodiversity conservation initiated between 1970 and 2019. Within this sample, we find that knowledge production about the Global South is largely produced by researchers in the Global North, implying a neocolonial power dynamic. We also find evidence of bias in reported ecological outcomes linked to lack of independence in scientific studies, serving to uphold narratives about who should lead conservation. We explore relationships in the sample studies between conservation initiative types, the extent of Indigenous Peoples’ and local communities’ influence in governance, and reported social and ecological outcomes. Findings reveal positive ecological and social outcomes are strongly associated with higher levels of influence of Indigenous Peoples and local communities and their institutions, implying equity in conservation practice should be advanced not only for moral reasons, but because it can enhance conservation effectiveness.

**Supplementary Information:**

The online version contains supplementary material available at 10.1007/s13280-024-02049-w.

## Introduction

In response to unabated global biodiversity loss, conservation actions are multiplying across the world (Watson et al. 2019; Gurney et al. 2023). Notably, the United Nations Convention on Biological Diversity adopted an ambitious Global Biodiversity Framework in December 2022, with targets to be achieved by 2030. These targets include a significant increase in the global area of land and sea under conservation measures to 30% (known as the “30 × 30 target”), effective restoration programmes for at least 30% of degraded ecosystems, and a reduction in the loss of areas of high biodiversity to close to zero (CBD 2022). Global meta-analyses of conservation initiatives’ performance have been interpreted to suggest that, on the whole, conservation actions have positive effects on biodiversity, and that funding for and implementation of current practices should therefore be expanded (Langhammer et al. 2024). However, this assumes conservation to be an apolitical exercise, whereas in practice site-level conservation interventions implemented around the world to protect, restore, or sustainably use nature vary greatly, and are highly political, complex, dynamic, and contested, affecting the lives of billions of people in profound ways (Pimm 2021). Unresolved questions remain over what forms of conservation work, particularly with regard to who should control and manage conservation, and on which values and knowledge systems conservation interventions should be based (Pascual et al. 2021; IPBES 2022; Pascual et al. 2023). Conservation monitoring data provide limited insights due to its geographic skew towards recording sites in the Global North, the types of conservation actions covered being primarily associated with state and NGO-led protected areas, while social, governance, human rights, and power dynamics remain largely overlooked (UNEP-WCMC 2018; Ghoddousi et al. 2022). The increasing number and geographic coverage of scientific studies of conservation practice offer a potential body of knowledge to nuance what lies behind some of these gaps (Moon et al. 2019). However, this also raises questions regarding how academic knowledge about conservation practice is produced, by whom, and what the implications of any biases and limitations might be (Cook et al. 2013; Colloff et al. 2017).

Here we present an in-depth synthesis of (English-language) 662 peer-reviewed empirical studies of site-level conservation interventions worldwide that were established over 50 years (1970–2019), including a wide range of interventions involving diverse actors and institutions on the ground. The sample of studies used is not assumed to be representative of global conservation practice or trends over time because the locations, initiatives studied, and questions explored are influenced by geographic biases and research trends.

Initially, we reflect on and analyse patterns within the sample, considering where and by whom knowledge about conservation is produced, and on how biases and potential conflicts of interest in conservation research might limit or shape its suitability to make inferences about conservation practice and its outcomes. With limitations, the dataset enables an exploration of the state of scientific knowledge about biodiversity conservation practice and provides a complementary alternative to the limitations of global conservation monitoring data, by providing insights into (i) the range and types of conservation interventions implemented, (ii) the extent of influence of Indigenous Peoples and local communities (IPs & LCs) in conservation governance, and (iii) factors influencing the ecological and social outcomes associated with conservation interventions.

### Conservation practice and the recognition of indigenous and local knowledge systems

Conservation interventions vary considerably, but often include one or a combination of the following: area-based or species protection, access or sustainable use regulations, livelihood support for local communities, financial incentives, education programmes, and customary management practices and local stewardship (Mace 2014; Apostolopoulou et al. 2021). The forms of governance employed in conservation also vary, according to who exercises control and through what institutions and interactions, including the extent of influence of IPs & LCs and their institutions relative to state, non-governmental organisations (NGOs), and private actors (Borrini-Feyerabend and Hill 2015). Inclusive and collaborative decision-making and respect for rights and diverse values are widely acknowledged in both science and policy as characteristics of good governance that underpin effective conservation (Ostrom 1990; CBD 2018; Agrawal et al. 2022; Chaplin-Kramer et al. 2023). IPs & LCs inhabit, manage and govern many land- and seascapes, resources and territories, in many cases having done so for generations, and have their own knowledge systems, comprising distinct worldviews, values, customary institutions and practices for relating to, conserving, restoring, or sustainably using nature (Corrigan et al. 2018).

There is great diversity among and between the world’s IPs & LCs and key distinctions are to be made between Indigenous, local and Western knowledge systems (Orlove et al. 2023). This means that studies pertaining to IPs & LCs and their roles in conservation require careful reflection on the values and the processes shaping the production of knowledge. Both conservation practice and science are political constructs which continue to be influenced by colonial logics. Among other things, they are often imbued with the Western values of organisations and actors in the Global North who dominate global conservation funding and decision-making (Adams and Mulligan 2012; Latulippe and Klenk 2020; Pascual et al. 2021). Conservation science influences practice profoundly, so it is important to reflect on the ways in which environmental orthodoxies and received wisdoms shape scientific findings and recommendations in order to help decolonise conservation science (Corbera et al. 2021).

The Global Biodiversity Framework explicitly calls for inclusive and equitable governance which recognises plural knowledge systems and the contribution made by IPs & LCs and their territories to conserving biodiversity (CBD 2022). There has been a progressive trend, particularly since the 1990s, towards the inclusion of indigenous knowledge in global conservation debates (Roué et al. 2022) and international policy (Brosius 2004; CBD 2018, 2022). Social objectives and equitable governance principles have also found their way into conservation science (Zafra-Calvo et al. 2019). This push for more inclusion and recognition of Indigenous and local knowledge systems has emerged through social movements calling for greater respect and recognition for IPs & LCs, long-term decolonisation processes and shifts towards community-based management in some parts of the Global South (Dietz et al. 2003; Brosius 2004; Ulloa 2013). It has also increasingly been supported by extensive theoretical and empirical research into the qualities of governance that support positive ecological outcomes of conservation (Borrini-Feyerabend and Hill 2015; Witter and Satterfield 2019).

Interventions that displace, exclude, or marginalise IPs & LCs are now widely acknowledged to be poor long-term conservation strategies, as they fail to allow for collaborative conservation efforts based on value and knowledge pluralism (Ostrom 1990; Springer et al. 2011; Schreckenberg et al. 2016; Bhola et al. 2021). However, the conservation initiatives implemented across the globe continue to fall most commonly under the control of states, NGOs, and private companies, even when overlapping with ancestral territories or community lands. Many interventions labelled as community-based or participatory forms of conservation have been evidenced to involve relatively minimal influence of IPs & LCs (Kumar 2005; Calfucura 2018; Galvin et al. 2018; Apostolopoulou et al. 2021). The slow pace of change has led to increased calls for a more profound decolonisation of conservation practice, and of knowledge production (Grove 2016; Corbera et al. 2021; Krauss 2021; Mabele et al. 2022).

Lands highlighted as priority areas for conservation—likely to form part of a new wave of conservation and restoration areas—are homes, territories, and ancestral lands to vast numbers of people, many of them Indigenous or traditional, local communities, ethnic and cultural minorities (Wilder et al. 2016). Indeed, nearly 1.8 billion people live in biodiversity hotspots across the world. Conservation interventions are well positioned to either include and empower or displace and marginalise these people, their customary institutions, values, and their traditional ecological knowledge (Allan et al. 2022).

There is mounting evidence that conservation in which IPs & LCs play an influential or central role is related to more positive social-ecological outcomes (Brondizio and Tourneau 2016; Schleicher et al. 2017; Dawson et al. 2021; Huber et al. 2023). Accordingly, the definition of protected and conserved areas has been broadened to include some locally led interventions as Other Effective Conservation Measures (OECMs), and efforts are ongoing to augment the inclusion of Indigenous territories, the majority of which are located in the Global South, within global conservation monitoring databases (Gannon et al. 2019). Yet, the World Database on Protected Areas is highly geographically skewed, with approximately 80% of sites recorded, as of 2018, occurring in Europe and North America (UNEP-WCMC 2018). Whatever the reasons for this weak representation of the actual global distribution of conservation efforts and of different forms of interventions within monitoring data, these conspicuous gaps make it very difficult to ascertain the effect of progressive policy principles for inclusion and recognition of IPs & LCs on conservation practice, and the relative effect of different governance regimes, including IP & LC led initiatives, on conservation outcomes.

The data collected for global protected and conserved area monitoring have been primarily concerned with management effectiveness indicators for protection, enforcement, and use of financial resources. The effectiveness of conservation interventions, particularly in protected areas, has been shown to be partly influenced by factors including funding levels (Coad et al. 2019), management effectiveness factors such as planning and administration systems (Powlen et al. 2021), as well as national political factors supporting or compromising quality of conservation governance (Eklund et al. 2011). However, the lack of information officially and systematically recorded about governance dynamics and the social impacts of conservation, alongside the limited spread of locations and different types of governance, has precluded more nuanced analysis of the full range, and distribution, of conservation practices and associated ecological and social outcomes (Moreaux et al. 2018; Ghoddousi et al. 2022).

The increasing number of interdisciplinary scientific studies of conservation across the world represents a key body of knowledge through which to explore these questions, as it covers social and institutional aspects that are lacking in formal monitoring data as well as ecological status and trends. To date, few studies have taken stock of conservation practices and outcomes at an international scale, with most of those limited to specific intervention types or comparisons between contrasting forms of governance. Redford et al. (2003) performed one of the few global reviews of conservation practice and identified 21 different conservation approaches, though only considered approaches implemented by international non-governmental organisations (NGOs) from the Global North. Zhang et al. (2023) reviewed various types of literature comparing the effectiveness of protected areas managed by states and areas managed by IPs & LCs. Garnett et al. (2018) conducted a spatial analysis revealing the global extent and conservation importance of Indigenous lands, while Dawson et al. (2021) undertook a global review of empirical studies to compare the reported impacts of locally versus externally controlled conservation interventions.

Several reviews of academic or grey literature, and large-scale data analyses about conservation practices and outcomes have provided evidence on specific regions, ecosystems, conservation policies, or intervention types. These include community-based conservation projects (Brooks 2016), integrated conservation and development programmes (ICDPs) (Wells and McShane 2004), terrestrial and marine protected areas (Oldekop et al. 2016; Ban et al. 2019), forest conservation interventions (Börner et al. 2020) and governance across selected countries (Persha et al. 2011) including those in the Amazon (Schleicher et al. 2017). All of these studies highlight that governance quality plays a key role, and collectively they provide compelling evidence of a relationship between the extent of inclusion, respect for rights and authority of IPs & LCs, and the achievement of positive social and ecological outcomes.

## Materials and methods

**Fig. 1:**
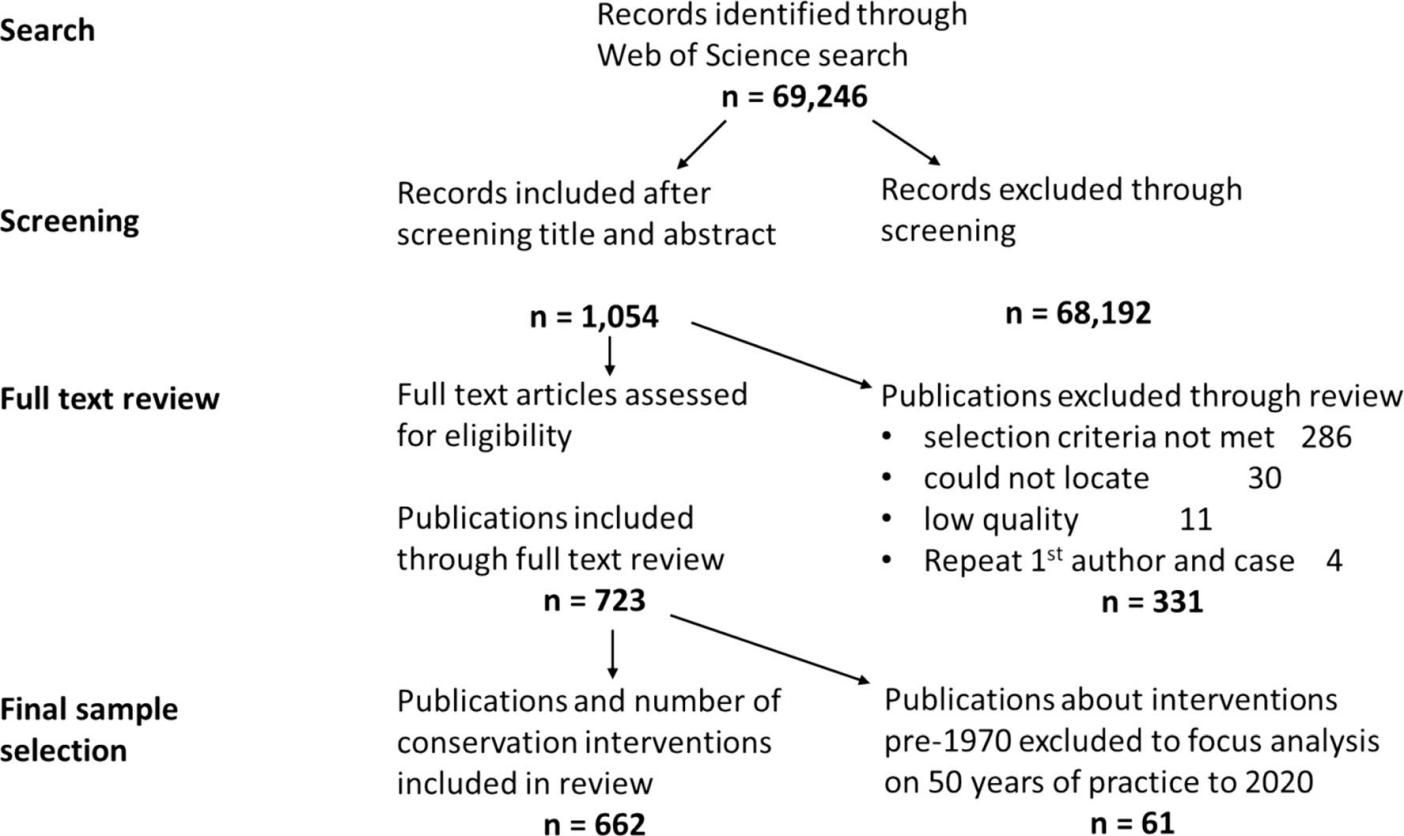
Flowchart of sample selection process leading to eventual sample size of 662 publications

We synthesise the results from the analysis of a sample of 662 studies (Fig. 1 depicts the sampling process) that describe and explore conservation efforts at a single site, i.e. comprising a particular landscape, ecosystem, habitat, or socially or administratively defined single area of conservation interest. The pool of studies was obtained through a keyword search, in English, on Web of Science, which returned 69 246 publications (See Supplementary Text S1). Following a pilot screening phase using 100 articles to test and refine the review protocol (Supplementary Table S1), a first screening of titles and abstracts was conducted between March 2020 and March 2021 by four of the authors using the open-access machine learning assisted software Colandr, which facilitates ordering of the sample publications by relevance. Among other things, Colandr enables selection of relevant publications from a large sample to become a realistic endeavour, as a reasoned cut-off rate can be applied so that only a small percentage of the entire sample need to be screened (Cheng et al. 2018). Titles and abstracts were screened and the machine learning software began to place the most relevant publications first. This ordering for relevance helped our inclusion rate to rise, reaching a peak of 18% included, for the 1000 publications screened between 3000 and 4000 (Figure S1). We set a cut-off rate to end the screening process of 3% included in the previous 1000, as a falling inclusion rate would suggest that the next 1000 would yield less than 30 additional papers. We reached this rate after screening 11 100, or 16% of the total number of screened studies. A sample of 1054 publications had been included by this stage (Fig. 1), and the steep decline in the inclusion rate suggested this was a pragmatic and justifiable cut-off to balance researcher time and sample size (Figure S1). The criteria for inclusion applied during screening were: (1) the study is about biodiversity conservation; (2) the study describes a deliberate intervention; (3) the study provides empirical evidence; (4) the conservation intervention takes place in a defined single site (studies based on multiple separate areas, or entire regions or countries comprising many ecosystems and landscapes were excluded) through a defined actor, group or organisation; (5) the study identifies a discernible conservation aim (whether the primary objective or not), and; (6) the study identifies a discernible conservation approach (Supplementary Information, Table S1).

We sought to include a range of conservation actions driven by a range of actors and we included any conservation-oriented initiative, as long as the criteria above were sufficiently met and described in the published study. The included types of conservation initiative spanned land, resource, habitat or species management, area protection, livelihoods and tourism programmes, sustainable use regulations or norms, financial incentives, compensation, education and capacity building, as well as local stewardship and traditional or customary management and practices.

Prior to data extraction, we conducted a training session and detailed collective coding exercise with ten papers to establish consensus about coding criteria and harmonise coding practices between eight of the authors and to refine the datasheet. Each case was then coded by a single researcher between March 2021 and March 2022, though the lead author worked with each of the coders to address any ambiguities about inclusion, categorisation, or data recording. The same exclusion criteria were applied during full review, with additional quality control to exclude papers lacking clear and appropriate methods, which resulted in a further 331 publications omitted (Fig. 1). For the analysis presented here, we also removed 61 cases about conservation interventions initiated before 1970, as they were spread very thinly back to 1800, and we decided to focus the analysis more concretely on the last 50 years of conservation practice, through the remaining 662 publications, each representing a conservation case study (Fig. 1). The data extraction from the final sample of 662 enabled us to capture and synthesise the study site and lead author locations and affiliations, different types of interventions, initiatives or practices being implemented, the extent of influence of IPs & LCs in conservation governance, and the associated social and ecological outcomes (Table 1).

**Table 1:** Description and definition of main variables and categories used in the analysis for the systematic review (for additional details on data extraction see Supplemental Appendix S1). The asterisk (*) indicates that the sample size was reduced as some publications did not sufficiently describe the variable of interest

Variable (sample size)	Categories (% of sample)	Definition
Type of conservation intervention (*n* = 662)	Species/ecosystem protection or restoration (15%)	Only area-based protection, ecosystem restoration or species/habitat regulations
	Financial incentives and compensation (6%)	Financial incentive instrument or compensation scheme for IPs & LCs (in addition to any in first category)
	Livelihoods, tourism, and capacity building (44%)	Conservation measures and/or projects with IPs & LCs regarding livelihoods, development, tourism, education, or capacity building (in addition to any from first category)
	Conservation through local stewardship (35%)	Conservation measures involving active management or enforcement role for IPs & LCs (in addition to any from the preceding categories)
Extent of IP & LC influence in conservation governance (*n* = 590*), as indicated by authors of sample publication in their description of governance	No IP & LC involvement (23%)	IPs & LCs and customary institutions were not involved in conservation decision-making
	Partial IP & LC involvement (56%)	IPs & LCs have some influence over conservation decision-making at some stage through a degree of participation, responsibility, or collaboration
	Locally led (21%)	IPs & LCs control or have the most influence over conservation decision-making and relevant customary institutions are a recognised part of governance
Ecological outcomes (*n* = 235*)	Positive (51%)	All ecological outcomes reported in the study are clearly positive
	Mixed (34%)	Outcomes reported are not clearly unidirectional. Trade-offs found, involving gains and losses in ecological indicators, or spatially, or temporally
	Negative (15%)	All ecological outcomes reported in the study are clearly negative
Social outcomes (*n* = 321*)	Positive (22%)	All social outcomes reported in the study are clearly positive
	Mixed (57%)	Outcomes reported not clearly unidirectional. Trade-offs noted between aspects of wellbeing, social groups, spatially or temporally
	Negative (21%)	All social outcomes reported in the study are clearly negative
Period in which the current form of governance was initiated (*n* = 662)	Multigenerational cases (10%)	Study describes IPs’ & LCs’ conservation-oriented customary institutions that have endured for multiple generations
	1970–1979 (7%)	The initiation year of the intervention, as reported in the reviewed publication
	1980–1989 (11%)	
	1990–1999 (28%)	
	2000–2009 (33%)	
	2010–2019 (11%)	
Institutional affiliation of study lead author (*n* = 662)	Global North (64%)	The institution to which the lead author is affiliated is in the Global North
	Global South (36%)	The institution to which the lead author is affiliated is in the Global South
Potential conflict of interest between research and intervention (*n* = 662)	Potential conflict of interest identified (14%)	One or more of the study authors declare a role in the conservation intervention; one or more authors are affiliated to an organisation involved in the intervention, or; the study was funded and supported by organisations involved in the intervention
	No potential conflict of interest identified (86%)	None of the above

To address the question of who produces conservation science (in English-language journals) and about which places, we explored the affiliations of lead authors of the studies and categorised them by country, continent and Global North or South (See Text S2 for definition used), and did likewise for the locations of case studies. To identify any potential conflicts of interest between the research behind the published studies and the conservation interventions being studied, we captured the affiliations of lead authors and also any funding declared as having supported the study, and noted where these overlapped with the conservation intervention of focus or whether any given interest was declared explicitly (Text S2). Following coding of all the cases, quality control and harmonisation of formatting for data entered was conducted by the lead author.

To detect potential temporal trends in the types of conservation interventions studied, the extent of IP & LC influence in conservation governance across those cases, and the social and ecological outcomes reportedly associated with them, we conducted Mann–Kendall tests in the package “Kendall” (McLeod 2022) in R Version 4.2.1 (R Development Core Team 2009), with each variable category as independent trendline. This was in part a data exploration exercise to inform the role of time as a variable in the regression analysis which followed. We did not assume this sample of empirical studies would indicate trends that are representative of changes in global conservation practice, in part because the locations selected and the trends in characteristics of interventions studied may be influenced by researcher interests and reporting. However, if the trend analysis highlighted any significant shifts in intervention types studied, the extent of IP & LC influence or outcomes reported, this could indicate a possible shift to be explored further and corroborated. Additionally, our literature search and screening led to the inclusion of 64 cases about multigenerational customary forms of conservation governance, involving forms of long-term stewardship by IPs & LCs and high levels of local control over natural resources. This subsample contains relevant information regarding the forms of knowledge, management, and governance systems. However, they could not be included within the trend analysis because no year could be assigned to the start of those interventions.

We recognise the important distinction between Indigenous Peoples and different types of local communities, in terms of their rights, roles in and contributions to conservation, their historical experiences and impacts, and the distinction between their knowledge systems (Orlove et al. 2023). For this systematic review, however, we included a wide range of types of people and communities under the umbrella acronym ‘IPs & LCs’. This is primarily driven by the sampled publications, which detailed impacts upon and the extent of influence of different Indigenous Peoples and non-Indigenous local communities, yet in many cases treated them as a single group of affected communities, or did not sufficiently detail their identities, histories, values, institutions or extent of political recognition to enable accurate distinctions to be made, particularly between traditional local communities and non-traditional local communities.

Ecological outcomes were reported in 235 of the 662 cases, through: biophysical data (22%); data on human actions impacting biodiversity (e.g. logging in forests, or trawl fishing) or perceptions about ecological outcomes (42%) or; both (36%). Social outcomes were reported in 321 of the cases, through: material social impacts such as change in income (10%); material outcomes plus an additional element, such as the extent of influence over decision-making or political empowerment (56%) or; an assessment giving attention to a range of possible material, social, cultural and political impacts and outcomes, which we refer to as a holistic social assessment (34%). 118 studies reported both social and ecological outcomes, meaning that 438 of the 662 publications reported either social or ecological outcomes or both, while the remaining 224 cases reported neither, and were therefore omitted from the outcomes analysis. The authors of studies not presenting data on outcomes focused on many different aspects including governance, values, knowledge, social dynamics, attitudes towards or perceptions of aspects of conservation other than outcomes.

We applied ordinal logistic regressions with ecological and social outcomes as response variables to model the relationships to the following explanatory variables: the type of intervention; the extent of influence of IPs & LCs in conservation governance; the lead author’s affiliation with an institution located in the Global South or North; the location of the conservation intervention in the Global South or North; and any identified potential conflict of interest due to the study´s funding or author affiliations being connected to the intervention in question. The 64 cases of multigenerational customary forms of governance were included in the sample for the regression analysis. The response variables for social and ecological outcomes were initially coded as negative, mixed, or positive and recoded ordinally as 0, 1, and 2, respectively, for analysis. Analyses were conducted with the package “ordinal” (Christensen 2023) and presented using the package “stargazer” (Hlavac 2022) in R Development Core Team (2009).

Prior to running regression models, we performed Cramer V tests to ensure the explanatory variables were not strongly associated with one another. We interpret association Φ-values of less than 0.5 to indicate a moderate association. All association values were below 0.5, with most exhibiting weak associations through Φ-values of less than 0.2. The two exceptions were the lead author affiliation and the study location (Global North or Global South), which were moderately positively associated (Φ-value = 0.293), and the intervention type and extent of influence of IPs & LCs in governance, which also showed a positive association (Φ-value = 0.499). The types of intervention such as stewardship, implicitly involve some level of influence of IPs & LCs in management, which we expect in most cases to be reflected in governance processes by at least partial involvement. Likewise, initiatives solely based on protection of an area or species are likely to involve lower levels of influence of IPs & LCs. However, the moderate association shows that these are not highly correlated through a Φ-value from 0.6 to 1, and so we took this as indication that both variables could be included within the regression models.

## Results

### Knowledge production in conservation science

**Fig. 2:**
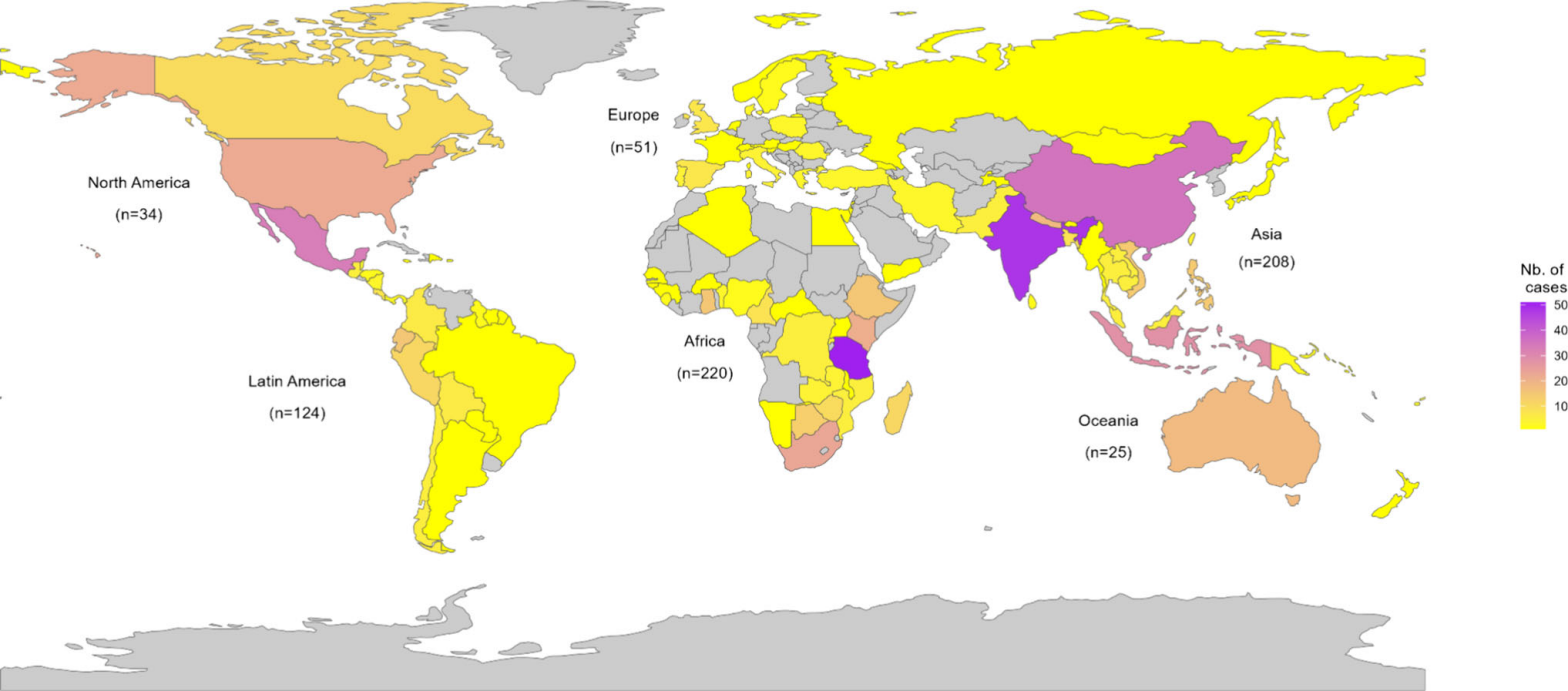
Map showing the number of reviewed cases of site-level conservation by country

From the analysed cases, the sites of conservation interventions were spread across 102 countries (Fig. 2), primarily located in the Global South (83% of total cases): Africa (*n* = 220 across 30 countries), Asia (*n* = 208, 24 countries) and Latin America (*n* = 124, 18 countries), with Europe, North America, and Oceania making up the remaining cases (*n* = 110 and 30 countries).

Ecosystems in which those interventions took place include forests (*n* = 290), coastal or marine realms (*n* = 106), rivers or wetlands (*n* = 78), savannah or dry forests (*n* = 65), mountains (*n* = 49), and grasslands, cultivated land, drylands, and other ecosystems (*n* = 74 combined).

**Fig. 3:**
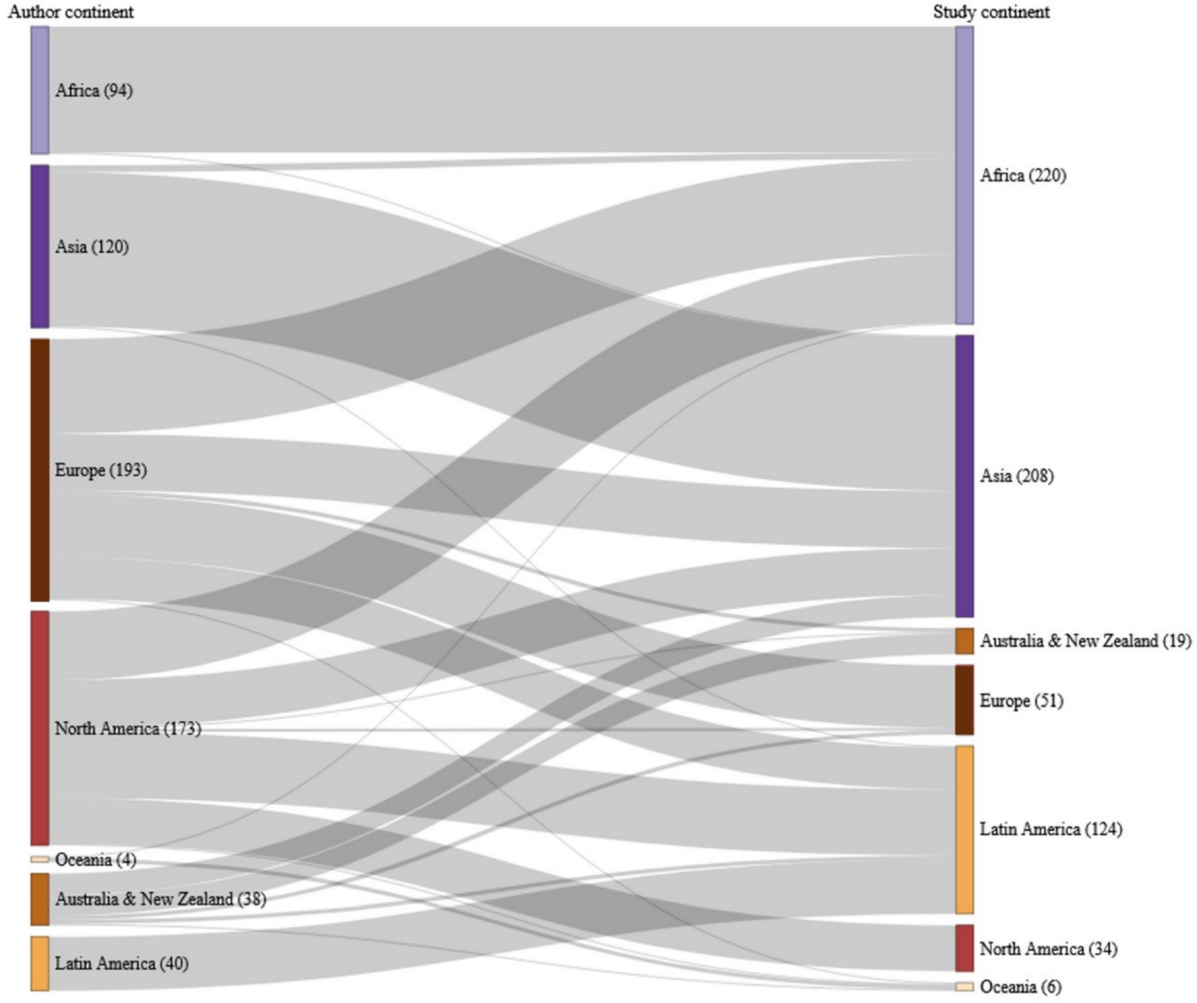
Origin of lead author affiliations relative to case study locations, by continent or group of countries, with sample sizes shown in brackets

**Fig. 4:**
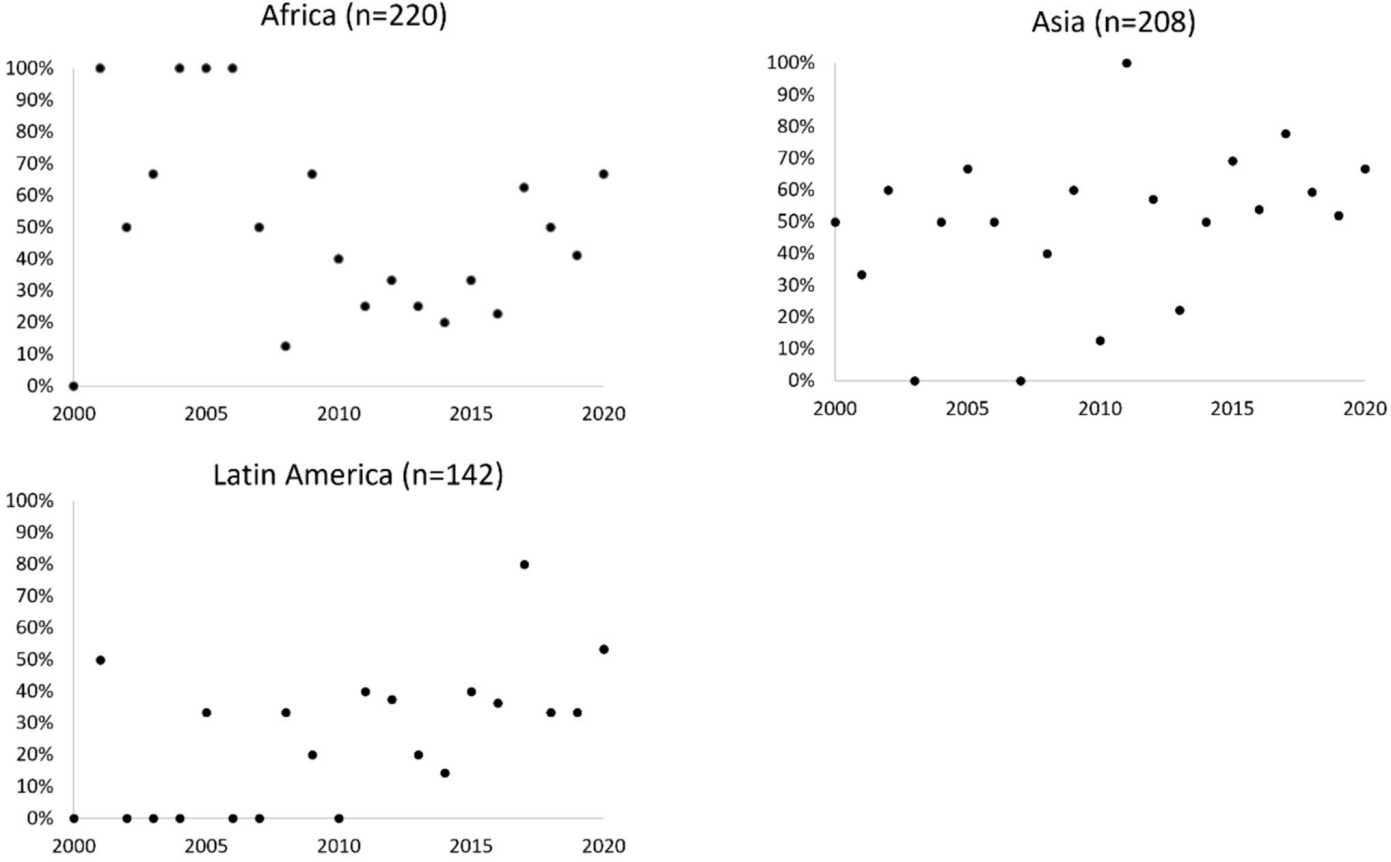
Proportion of the case studies of conservation interventions in Africa, Asia, and Latin America written by lead authors affiliated to organisations in the same continent (total 93 out of 220 for Africa, 114 out of 208 for Asia, and 40 out of 124 for Latin America). Data are displayed by year ranging from 2000 to 2020

Our results reveal a stark inequality in the production of knowledge between Global North and Global South. Most studies focused on locations in the Global South (83%); however, most of the lead authors of those studies were affiliated with research institutions in the Global North (64%), particularly from North America and Europe (Fig. 3). In contrast, not a single lead author from Asia, Africa, Latin America, or Oceania led a study of a conservation initiative in Europe, North America, Australia, or New Zealand. The proportion of conservation initiatives in Africa, Asia, and Latin America that were the subject of studies led by authors from those respective continents appears to have increased slightly in the most recent years, particularly from 2010 to 2020 (Fig. 4). The relationship is unclear for the period 2000 to 2010 for all three continents, possibly because the sample sizes were relatively small for those years, with commonly five or less publications per annum.

The results from the regression analysis suggest that when studies are led by authors from the Global South, there is a weak positive influence on reported ecological outcomes (*p*-value < 0.1). When case studies were located in the Global South, we found a significant negative influence on reported ecological outcomes (*p*-value < 0.05).

**Fig. 5:**
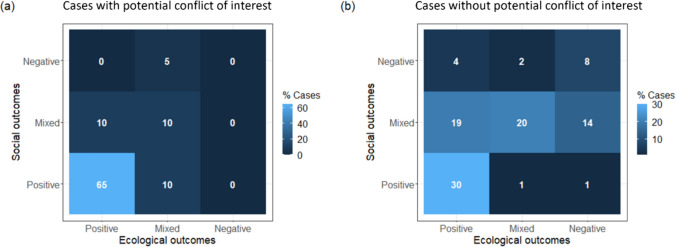
Comparison of the combination of social and ecological outcomes reported **a** through studies with a potential conflict of interest and **b** those without

We identified 94 publications (14% of all reviewed studies) with a potential conflict of interest (see also Table 1 and description of methods above). The data suggest that studies exhibiting a potential conflict of interest through their funding or author affiliation are far more likely to report both better ecological and social outcomes (regression analysis presented later in results shows this to be statistically significant, *p* < 0.01, Table 2). Concurrently positive social and ecological outcomes were reported in 65% of cases with a potential conflict of interest, compared to 30% of cases in which no conflict of interest was identified (Fig. 5). Crucially, the proportion of cases exhibiting a potential conflict of interest and reporting jointly positive social and ecological outcomes is consistent across categories for the level of influence of IPs & LCs (ranging from 50 to 64% and highest for those with partial involvement). We have no specific grounds to question the findings of any particular study. However, across all case studies with an identified potential conflict of interest, the collective propensity to record positive outcomes equally across governance categories contrasts markedly with the studies with no identified conflict of interest, which reported joint positive outcomes in 0% of cases with no IP & LC involvement, just 19% for those with partial IP & LC involvement and ranging up to 59% for the locally led category. Therefore, it appears prudent to also run the regression analysis after removing the cases with a potential conflict of interest from the sample. Removing these cases reinforced (though did not substantially alter) the significantly positive influence that leadership by IPs & LCs (having primary control over an initiative) has on both the reported ecological and social outcomes (*p*-value < 0.01, Supplementary Information, Table S2).

**Table 2:** Outputs of the ordinal regression models to analyse the factors influencing the social and ecological outcomes of conservation practices. The numbers displayed represent coefficient estimates and those in brackets are standard errors. * denotes *p*-value < 0.1, ** denotes *p*-value < 0.05, and *** denotes *p*-value < 0.01. Supplementary Table 2 shows the outputs of the ordinal regression models after omitting sample cases that identified a potential conflict of interest between the study’s funding or author affiliations

Explanatory variable	Dependent variable	
	Ecological outcomes	Social outcomes
Intervention type: Incentives and compensation	1.355* (0.823)	0.302 (0.638)
Intervention type: Livelihoods, tourism, and capacity building	− 0.439 (0.600)	0.580 (0.479)
Intervention type: Stewardship by IPs & LCs	0.225 (0.659)	1.098* (0.561)
Extent of IP and LC influence: Partial involvement	0.539 (0.480)	1.051*** (0.334)
Extent of IP and LC influence: Locally led	2.246*** (0.657)	2.587*** (0.497)
Lead author from the Global South	0.571* (0.343)	− 0.229 (0.265)
Case study from the Global South	− 1.121* (0.505)	− 0.480 (0.441)
Potential conflict of interest in authorship or funding	1.339*** (0.428)	1.880*** (0.417)
Number of observations (describing governance as well as ecological or social outcomes, Table 1)	182	294
Log likelihood	− 153.725	− 237.736

### Temporal trends in conservation initiatives, governance, and outcomes

**Fig. 6:**
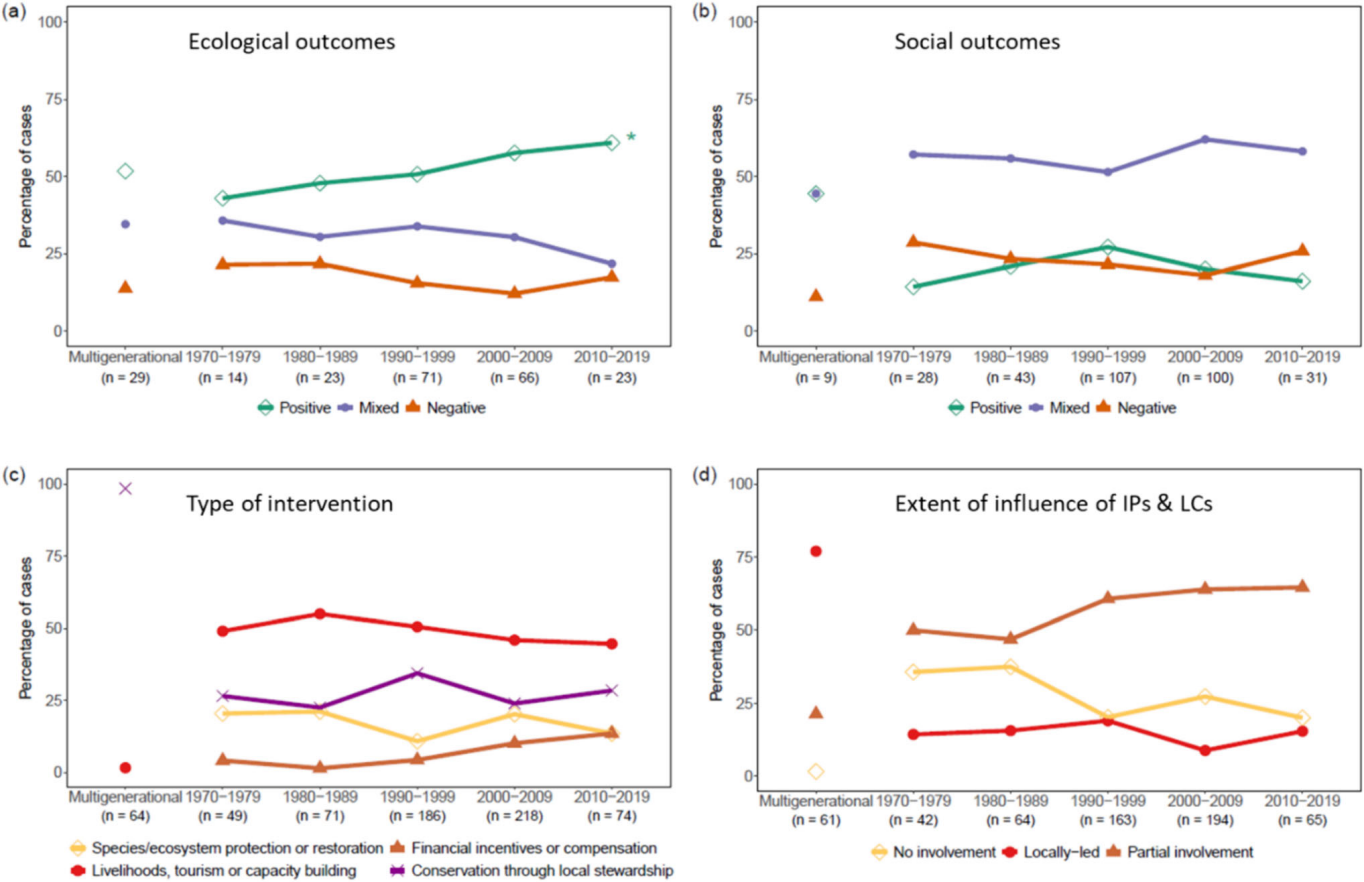
Temporal trends in **a** ecological outcomes, **b** social outcomes, **c** intervention types, and **d** extent of influence of IPs & LCs in conservation governance, as recorded in the 662 published studies reviewed, and displayed as a proportion of the 662 conservation interventions they describe. Note that because the cases based on multiple generations of customary institutions have no discernible year of inception, they could not be grouped within a decade so are displayed separately from the depicted trends and labelled as ‘multigenerational’. The asterisk (*) indicates a significant Mann-Kendal trend test for positive ecological outcomes (tests for all other variables are non-significant)

The changes in the conservation types observed and outcomes recorded over the five-decade (1970–2019) period from the analysed sample of published studies are shown in Fig. 6, broken down into ecological and social outcomes (Table 1, Fig. 6a, b), the type of conservation intervention (Table 1, Fig. 6c) and the level of influence of IPs & LCs in governance (Table 1, Fig. 6d). Initiatives focused solely on protection or restoration formed less than a quarter of cases in any decade, with the largest proportion (44%) involving livelihoods, tourism, or capacity building projects followed by initiatives involving management through the stewardship of IPs & LCs (35%). No clear or significant temporal trends were noted over the five decades; however, initiatives based on financial incentives or compensation for IPs & LCs increased steadily over time as a share of the studies sampled.

Across the five decades, the share of studies reporting positive ecological outcomes increased significantly over time (Fig. 6a, Mann–Kendall trend: tau = 1; 2-sided *p*-value = 0.027) while trends were non-significant for all other variables. This increasing trend was such that more than half of the studies documenting interventions initiated between 2000 and 2009 reported positive ecological outcomes, rising to 61% for interventions starting between 2010 and 2019. It is important to note though that the trend does not necessarily demonstrate a general improvement in the effectiveness of conservation over time, for example, because it might reflect a tendency for scientists and academic journals to publish studies where positive outcomes are more apparent than not. A far higher proportion of studies reported positive ecological outcomes than positive social outcomes (Table 1, Fig. 6a, b).

### Factors influencing outcomes of conservation practice

With regard to the type of conservation intervention, the results from the regression analysis suggest that the interventions associated with stewardship by IPs & LCs have a positive but weak effect on social outcomes relative to interventions focused solely on nature protection or restoration (*p*-value < 0.1). Interventions involving incentives or compensation for IPs & LCs exhibit a weak positive effect on ecological outcomes (*p*-value < 0.1).

The extent of influence of IPs & LCs in conservation governance shows a greater influence on both social and ecological outcomes. Specifically, the data suggest that the local leadership of conservation by IPs & LCs has a significant influence on the likelihood of achieving more positive ecological outcomes (*p*-value < 0.01) and more positive social outcomes (*p*-value < 0.01), compared to interventions where IPs & LCs are not involved in conservation governance and practices (Table 2). In addition, a partial involvement of IPs & LCs in governance also shows positive influence on social outcomes relative to cases where IPs & LCs are not involved at all (*p*-value < 0.01), though the influence on ecological outcomes is not statistically significant (see Table 2). To provide an illustrative example from the sample studies, Araos et al. (2020) detail how a social movement drove the establishment of the Los Lagos Indigenous Marine Areas in Chile in 2012, which produced positive social and ecological outcomes in reaction to, and relative to, the rapid degradation caused through widespread commercial Atlantic salmon farming. In a contrasting case, Kusumawati and Visser (2014) detail how the Berau Marine Conservation Area in Sulawesi, Indonesia, had to be annulled only five years after its 2005 inception due to the lack of local communities’ involvement and displacement of customary institutions.

## Discussion

Our systematic review, resulting in the analysis of 662 published empirical studies of site-level conservation, enables reflection on aspects of knowledge production in conservation science. The issues and inequalities raised in the analysis imply the need for exploratory and decolonial approaches to studying conservation, considering diverse actors, plural knowledge systems, experiences, and practices beyond Western scientific logics and narratives. Although we are limited by the research approaches within the sample publications, we developed a broad, novel dataset containing a wide array of conservation initiatives and comprising a relatively complete spectrum of possible power relations and allocation of rights and responsibilities (see Coolsaet and Dawson 2023). The role of IPs & LCs ranges from exclusion to partial involvement as participants, stakeholders, or partners, and through to primary or complete control over governance of an ecosystem or landscape.

Our analysis of the social and ecological outcomes recorded for those widely differing forms of conservation reveals that local control and recognition of IP & LC institutions are strongly associated with more positive outcomes for people and nature. This is in line with findings from a growing number of studies at different scales and employing varying methods, for Indigenous territorial governance particularly, but also for customary and more contemporary local institutions (Persha et al. 2011; Corrigan et al. 2018; Börner et al. 2020; Benzeev et al. 2023; Zhang et al. 2023).

Our sample captured far more studies from the Global South than North. Our search terms were designed to be inclusive and not discriminatory. Our inclusion criteria demanded a depth of focus to detail the objectives and approach being taken in a locality. It is possible that the design and implementation of conservation receive more research scrutiny in the Global South (Barrett et al. 2013; Murdock 2021). We found relatively negative ecological outcomes reported from the Global South, perhaps due to higher rates of land-use intensification and change or greater tradeoffs or opportunity costs of conservation, and this provides a potential justification for the disproportionate scientific attention. Certainly, many North American and European researchers study cases in the Global South rather than within their own country or continent, with negligible exchange in the other direction. Decolonial scholarship has long highlighted the tendency of reducing Southern contexts to the “empirical” or the material (see e.g. de Sousa Santos 2015), and this persistent inequality, reflected also in the publishing system, research funding streams and global economic and geopolitical dynamics, may explain the bias towards Global South case studies by Global North researchers. Additionally, disciplinary differences may contribute—many of the studies of conservation in the Global North excluded during abstract screening were very specific biological or economic studies which failed to provide sufficient depth or focus on the conservation aims and approach to be included. Whatever the reasons, specific efforts are required to increase Global South-led conservation science through better funding availability, publishing options, and leadership roles in international partnerships to researchers and institutions in the Global South.

Our review highlighted a prevalence of independence issues in empirical studies. Within the analysed sample, almost one in seven studies exhibit a potential conflict of interest between author affiliations or research funding with the conservation intervention. Relative to the remainder of the sample, this subset has a significant direction of bias towards reporting more positive social and ecological outcomes (Figure S3, Table S2), with proportionately more success reported for initiatives excluding or only partially involving IPs & LCs relative to studies without an identified conflict of interest. Several studies have highlighted the lack of work about failures in conservation science and the negative impacts this has on progressive change inspired by lessons learned (Catalano et al. 2019; Chambers et al. 2022). Mostly, the biases we identified may serve to exaggerate the success of mainstream practices and support the status quo in conservation governance. For example, this can imply that the achievement of positive outcomes is possible through factors like funding allocation, regardless of whether an intervention is exclusive of or led by IPs & LCs, whereas the leadership of IPs & LCs is clearly highlighted as the primary influence on conservation effectiveness by independent studies. The reporting bias may also arise because the researchers actively or subconsciously seek to placate organisations and funders, or only publish work if they have a positive message to communicate (Pillay et al. 2020). Whatever the reason, conflicts of interest as well as funding and resource inequalities are clearly an issue within conservation science, deserving targeted scrutiny due to the potential to reinforce unequal power relations, to obscure lessons and misguide policy and practice.

Our analysis of a wide range of site-level conservation initiatives over five decades reveals that conservation interventions led by IPs & LCs are reported through empirical research to produce significantly better ecological and social outcomes than those which either exclude them or enable only partial involvement (Table 2). The extent of control by IPs & LCs, and recognition of their customary or local institutions and knowledge, appears to be a key characteristic of governance influencing conservation success, and therefore a governance quality that all conservation actors should engage in working collaboratively towards. The data and findings relate to any form of intervention, whether protected areas, restoration, sustainable use, or incentive schemes, irrespective of the mix of stakeholders involved, and regardless of the region or type of ecosystem where conservation takes place.

These findings have implications for how to pursue the ambitious 2030 targets for conservation and restoration in the CBD’s Kunming Montreal Global Biodiversity Framework (CBD 2022). The principles of equitable governance and recognition for IPs & LCs are already well aligned with standards in conservation conventions, policies, organisations, and programmes (CBD 2018, 2022), but a disconnect has endured between this rhetoric and the practices being implemented on the ground (Tauli-Corpuz et al. 2020). Our findings imply that if IPs & LCs play only a marginal role in various conservation projects initiated to meet the Global Biodiversity Framework’s targets for 2030 and beyond, there is considerable risk they will have limited success in curbing biodiversity loss, because conservation dominated by external actors lacks the qualities of governance most appropriate to generate positive outcomes for people and nature (Reyes-García et al. 2022).

The exclusion or minimal role of IPs & LCs as consultees or stakeholders, and inattention to local tenure security and institutions, is entrenched in many conservation practices, alongside ideas that conservation expertise lies with external actors (Woodhouse et al. 2022). While we can envision cases where decision makers would argue and perceive that the exclusion, displacement, or marginal roles of IPs & LCs are “necessary”, *e.g.* to protect rights of nature, neither policy norms nor scientific evidence supports such practices and offers no justification for normalising exclusionary forms of conservation or situations where states and external actors dictate decision-making (Rights and Resources Initiative 2020). In many locations around the world, IPs & LCs are those who take on considerable burdens and risks to act as environmental or rights defenders protesting against environmentally harmful laws, policies, projects, and actions (Boyd and Keene 2021; Cariño and Ferrari 2021).

To drive a change in conservation effectiveness and social justice, the character and quality of governance, at multiple scales, must become a guiding objective (Pascual et al. 2022). Conservation governance is not a simple choice between types based on who has ultimate control—actors’ interests vary widely, conservation objectives almost always include social as well as ecological aims, and there are plural knowledge systems to collaborate across, meaning that governance processes are complex, dynamic, and negotiated (Pascual et al. 2021, 2022; Droz et al. 2023). It is important to emphasise that rapid switches in governance types and power relations, for example, from state control to Indigenous or local autonomy, are unlikely to realise a rapid upturn in results without considerable support to build, strengthen, or reinforce local and customary institutions. Rather, it is in the interests of all conservation actors to engage in collaborative efforts to pursue more equitable conservation by enhancing the roles and recognition of IPs’ & LCs’ authority in conservation governance—in policies, projects and in local actions and interactions—through strategies adapted to the social, political, and environmental context and the resources and institutional strengths of IPs & LCs who live there (Borrini-Feyerabend and Hill 2015; Armitage et al. 2020; Dawson et al. 2023).

These standards of governance do not only apply to entirely new interventions to meet the Global Biodiversity Framework targets for 2030, but equally apply to existing interventions, and to areas of importance for biodiversity which lie outside officially recorded protected and conserved areas. Crucially, supporting and enabling the positive impacts of Indigenous and locally led conservation require political and legislative transformations at national and sub-national levels in order to counter the structural barriers caused by state control over natural resources, land tenure, and other entrenched colonial power dynamics (Asia Indigenous Peoples Pact et al. 2022). Global progress in this direction is patchy—in the five years prior to 2023, policies supporting Indigenous territorial rights as pathways to conserve biodiversity have had impacts in Canada and New Zealand, but were concurrently eroded in other countries through political obstruction and discrimination, for example, in Brazil (Artelle et al. 2019; Karjoko et al. 2021). It is important to note that the historic injustices, extent of recognition or discrimination, relationships, forms of representation and pathways to change can be quite different for Indigenous Peoples relative to local communities and to traditional communities among them.

Our review analysis has some limitations. First, our focus on studies published in English leads to geographic bias. The large proportion of cases from Tanzania in particular has been noted in other studies and reflects the high number, diversity, and coverage of conservation interventions there (Riggio et al. 2019; Apostolopoulou et al. 2021). Second, English-language peer-reviewed literature shows bias towards work by researchers from the Global North and may overlook the work of academics, civil society researchers, as well as Indigenous scholars that better reflect non-western knowledge systems and issues relating to power, race, gender, and culture (Karlsson et al. 2007; Asase et al. 2022; Droz et al. 2023). Especially in Tanzania, the production of conservation knowledge is dominated by foreign academics over local scholarship (Mabele et al. 2023). Third, only a small proportion of peer-reviewed studies provide sufficient information about the aims, actors involved and approach associated with an intervention, as well as the associated social and/or ecological outcomes, which restricts the size of the sample, particularly for analyses of factors associated with certain outcomes. Based on the observed lack of holistic approaches to assess conservation governance and outcomes, more interdisciplinary studies and dedicated funding streams are required to enable improved and more holistic assessments of conservation practice.

We treated all IPs & LCs as a single group for our analyses, despite recognising the important distinctions between Indigenous Peoples, traditional local communities, and non-traditional local communities. The Intergovernmental Platform for Biodiversity and Ecosystem Services (IPBES) limits the definition of IPs & LCs to include communities who self-identify as Indigenous and hold distinct rights, in addition to local communities who maintain inter-generational connections to place and nature through customary values, institutions and practices—while emphasising that each category is very diverse as well as distinct in key ways (Watson et al. 2019). However, for this study we also included non-traditional local communities who do not hold customary, place-based values and related institutions, whether because they have experienced disruption to them, been displaced or they represent more recently formed communities comprising people from diverse places and with diverse identities. This latter group can nonetheless form and exhibit shared values, connections to place and nature, meaning they can experience various social impacts from conservation and also seek to contribute to, establish and revitalise institutions and practices for sustainable management (Bunch 2016; Murphy et al. 2019).

Indigenous Peoples were specifically mentioned as being affected by or involved in 24% of the 662 initiatives, though not always separately from local communities. The distinction was best described in studies of initiatives based upon Indigenous or local institutions, though much less so for those externally controlled and involving only participation by or exclusion of IPs & LCs. Future studies may develop greater understanding of the differences their disaggregation may mean for the relationships we found and the social characteristics and governance dynamics influencing them.

Finally, our relatively small sample size, while providing a fair representation of conservation science, may follow researcher preferences and research funding and publishing trends that are not representative of conservation practice more generally. This is especially true as most Indigenous and community conservation endeavours go unstudied by the western scientific gaze, take place with or without structural support, and consequently are published in reports as opposed to peer-reviewed journals (Asia Indigenous Peoples Pact et al. 2022). However, our dataset is still of sufficient size and breadth to capture a diversity of on the ground conservation interventions, governance types, and a wider range of outcomes than global conservation monitoring platforms cover, which enables a more exploratory reflection on the implementation of conservation over the long term, with novel insights for how best to pursue social and ecological goals in tandem.

## Conclusion

Our review has clear implications for both conservation science and practice: to pay greater attention to Global South perspectives and ensure they gain greater representation in the production of knowledge regarding biodiversity conservation practice and governance, and to pay attention to potential conflicts of interest between science and practice, which may reinforce common assumptions about who drives conservation success and act as a barrier to transformation towards Indigenous, local, and Global South leadership. Science and practice are closely linked and must both provide increased consideration to, inclusion of and collaboration across plural knowledge systems and diverse ways of knowing and conserving. This is in accordance with calls to place, empower, revitalise, and support Indigenous knowledge systems or local knowledge systems at the centre of conservation strategies with embedded objectives and interactions supporting decolonisation (Latulippe and Klenk 2020; Apostolopoulou et al. 2021; Corbera et al. 2021; Krauss 2021; Rodriguez 2022; Orlove et al. 2023).

Amid the rapid scaling up of conservation driven by the Global Biodiversity Framework 2030 targets, it is crucial that long-term evidence drawing from the full diversity of conservation efforts is used to guide necessary changes in practice, through collaborative efforts to enhance governance and conservation effectiveness. Critical scientific studies exploring and synthesising this evidence base (including this review) consistently find that to conserve nature most effectively, and to concurrently meet standards for IP & LC rights and social justice, conservation practice must take a step change to ensure IPs & LCs are empowered, recognised as authorities, and able to apply and revitalise their own knowledge and institutions to sustain both nature and people.

## Supplementary Information

Below is the link to the electronic supplementary material.

Supplementary file1 (PDF 793 KB)

## Data Availability

The dataset for this study is available at Zenodo 10.5281/zenodo.7688777.
