# Supplementary material for: Reviewing the science on 50 years of conservation: Knowledge production biases and lessons for practice
Source: Ambio. 2024 Jul 18;53(10):1395–413. doi: 10.1007/s13280-024-02049-w (PMC11383897; doi:10.1007/s13280-024-02049-w)
Supplement: Supplementary file 1 — Supplementary file1 (PDF 793 KB) [file 13280_2024_2049_MOESM1_ESM.pdf]

Ambio

Supplementary Information (*This supplementary information has not been peer reviewed)*

Title: Reviewing the science on 50 years of conservation: Knowledge production biases and lessons for practice

Supplementary Text S1.

Initial search string used in Web of Science Core Collection on 18th September 2020, identifying 69,246 publications.

((TS=(“conservation”) AND TS=(“biodiversity” OR “biological” OR “nature” OR “natural resource*” OR “ecosystem” OR “habitat” OR “wildlife”) AND TS=("governance" OR "protected area*" OR "conserved area*" OR "preserved area*" OR "wilderness area*" OR "reserve*" OR "park*" OR "resource management" OR "ecosystem management" OR "wildlife management" OR "habitat management" OR "restoration" OR "preservation" OR "protection" OR "species management" OR "alternative livelihood" OR "livelihood alternative" OR "livelihood* program" OR "incentive" OR "revenue shar*" OR "revenue-shar*" OR "ecotourism" OR "tourism" OR "conservation and development" OR "access and benefit-sharing" OR "certification" OR "compensat*" OR "payment* for ecosystem service*" OR "capacity-build*" OR "capacity build*" OR "training" OR "educat*" OR "stewards*" OR "sanctuary" OR "custodian" OR "guardian" OR "hunting ground" OR "tribal" OR "sacred" OR "spiritual" OR "indigenous people*" OR "community-based" OR "co-manag*" OR "decision-making" OR "decision making" OR "law*" OR "policy" OR "regulat*" OR "intervention" OR "institution*" OR "participation" OR "sustainable use" OR "sustainable utilisation" OR "sustainable utilization" OR "traditional knowledge")))

Supplementary Table S1. Criteria for screening at title and abstract level

Criteria for exclusion	Description
**Not conservation**	The paper is not about nature/biodiversity conservation
**No intervention**	The study is not related to a defined conservation 'act' and/or doesn't identify a certain actor (e.g. no studies describing only research except if it is part of a defined intervention).<br>**Note: Local, customary practices count as conservation actions. We do not intend intervention to mean there must be a project that has been implemented by a conservation-oriented organisation. Instead we consider conservation-oriented governance and management of any kind if described in sufficient detail.
**Not empirical paper**	Empirical case studies only (no discussion papers, no overviews, no reviews/synthesis, no future scenarios, no modelling).
**No clear location**	Exclude multi-site papers except if context and outcomes of 1 site are clearly discernible.<br>Inclusion requires not only details of a policy, program design, aims or conservation approach (which many papers provide at a national or regional scale) but clear details about how it has been implemented or applied in a defined location and context - beyond superficial understanding or envisioned governance.

**No clear target**	The paper does not identify a discernible conservation target, e.g. species conservation (single or multiple species), ecosystems & habitat conservation, ecosystem services or processes, landscapes & scenery, development/access/wellbeing/human rights, and not just ‘conservation’, or ‘biodiversity’.
**No clear approach**	The paper does not identify a discernible conservation approach and/or does not (appear that on full review it will) provide enough information about the approach being applied at that location to enable us to discern the key design features. Examples include area protection, land, resource, habitat or species management, livelihoods and tourism, financial incentives, compensation, education and capacity building, local stewardship, traditional practices, sustainable use.

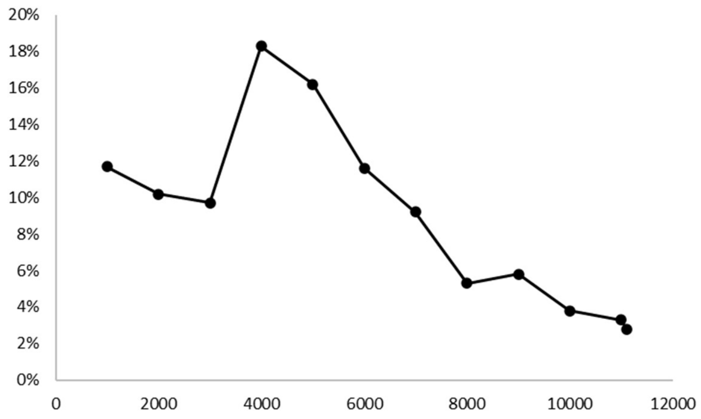

Supplementary Figure S1. Decision cut off for screening process in Colandr machine learning platform. The y-axis displays the percentage of publications included in the sample per previous 1000 abstracts and titles screened, and the x-axis displays the total number of publications screened. The cut-off point to end screening was selected as 3%, which was met after 11,100 of the 69,246 publications had been screened. The remaining 58,146 were therefore all excluded.

Supplementary Text S2: Coded variables for data extraction through full review

Bibliography and methodology section

- **study_id**: unique identifier of the study
- **title**: title of the study
- **year**: publication year of the study
- **authors**: authors of the study
- **name_journal**: name of the journal the study was published in
- **author_affiliation_type**: type of institution the lead author of the study works for (University/Academic; NGO; public/state/UN; think thank/research institute; private company; independent; other). Only lists first affiliation.
- **author_affiliation_country:** country of affiliation of the lead author. Only lists first affiliation.
- **author_continent**: continent of affiliation of the lead author. Only lists first affiliation.
- **author_north_south**: indicates whether the lead author is affiliated with an institution in the Global North (N) or Global South (S), as defined at https://unstats.un.org/unsd/methodology/m49/ (accessed 29th May 2022). Only lists first affiliation.
- **major_funder_ngo**: are any authors affiliated with a major conservation funder or organisation with an interest in the conservation intervention being researched (yes; no)
- **funding_source**: funding source of the paper (not of the conservation intervention)
- **conflict_of_interest:** overlap between the affiliations of authors, the funding source and the conservation intervention being researched (0 = no; 1 = yes)

- **study_country**: country where the intervention was studied
- **study_locality**: locality or region where the intervention was studied
- **study_continent**: continent where the intervention was studied
- **study_north_south:** indicates whether the conservation intervention was conducted in the Global North (N) or Global South (S), based on UN Stats definition.

Conservation target & intervention section

- **primary_ecosystem_type**: type of ecosystem of the intervention (Forest/Woodland; Savanna/Dry forest; Dryland/Semi-arid; Grassland/Shrubland; Rivers/Wetlands; Coastal/Marine; Mountain; Cultivated; Other)
- **name_primary_intervention**: name of the conservation intervention or program
- **year_primary_intervention**: year the conservation intervention or program started (single year; many years)
- **target_type_ecosystem**: the conservation intervention targets ecosystems or habitats (yes; no)
- **target_type_species**: the conservation intervention targets defined species (yes; no)
- **target_type_ess**: the conservation intervention targets ecosystem services or processes (yes; no)
- **target_type_humans:** the conservation intervention targets humans and livelihoods (yes; no)
- **description_primary_target**: short description of the primary conservation target
- **description_secondary_target:** short description of any additional conservation target (if applicable)
- **intervention_type_paca**: the conservation intervention takes places through protected areas or conserved areas (yes; no)

- **intervention_type_restoration**: the conservation intervention takes places through restoration (yes; no)
- **intervention_type_species_use**: the conservation intervention takes places through species protection or sustainable use measures (yes; no)
- **intervention_type_livelihoods_tourism**: the conservation intervention takes places through livelihoods support or tourism (yes; no)
- **intervention_type_incentives**: the conservation intervention takes places through incentives, compensation schemes, or market instruments (yes; no)
- **intervention_type_education**: the conservation intervention takes places through education or capacity building (yes; no)
- **intervention_type_stewardship**: the conservation intervention takes places through local/indigenous stewardship (yes; no)
- **description_primary_type**: short description of the primary conservation type
- **description_secondary_type:** short description of any additional conservation types (if applicable)

Governance section

- **study_covers_governance:** the paper describes governance processes (yes; no)
- **iplc_involved:** indigenous people and/or local communities are involved in the conservation intervention (no; yes, partially; yes, locally-led)
- **description_governance**: short description of the governance process and main actors involved
- **additional_notes:** notes and/or remarks about conservation intervention/governance

Outcomes section

- **ecological_outcomes_incl:** study describes ecological outcomes or conservation effectiveness (yes; no)
- **ecological_outcomes:** the reported ecological outcomes are positive, negative, or mixed (positive; negative; mixed; N/A)
- **social_outcomes_incl**: study describes social impacts or outcomes associated with conservation (yes; no)
- **social_outcomes:** the reported social outcomes are positive, negative, or mixed (positive; negative; mixed; N/A)

Supplementary Table S2. Outputs of the ordinal regression models to analyse the factors influencing the social and ecological outcomes of conservation practices. Sample cases were omitted from this model if the review identified a potential conflict of interest between the study's funding or author affiliations (see Table 1, main text). The numbers displayed represent coefficient estimates and those in brackets are standard errors. * denotes p-value<0.1, ** denotes p-value<0.05, and *** denotes p-value<0.01.

Explanatory variable	Dependent variable	
	Ecological outcomes	Social outcomes
Intervention type: Incentives and compensation	0.718	-0.050
	(0.897)	(0.676)
Intervention type: Livelihoods, tourism and	-0.889	0.499
capacity building	(0.676)	(0.493)
Intervention type: Stewardship by IP & LCs	-0.437	1.112*
	(0.753)	(0.593)
Extent of IP and LC involvement: Partial	0.692	1.204***
involvement	(0.522)	(0.351)
Extent of IP and LC involvement: Locally-led	2.790***	2.871***
	(0.726)	(0.529)
Lead author from the Global South	0.538	-0.310
	(0.381)	(0.288)
Case study from the Global South	-1.449***	-0.541
	(0.559)	(0.463)

Number of observations	146	260
Log likelihood	-127.015	-204.586
